# The Microbiota–Gut–Brain Axis in Behaviour and Brain Disorders

**DOI:** 10.3390/ijms24108460

**Published:** 2023-05-09

**Authors:** Daniele Lana, Maria Grazia Giovannini

**Affiliations:** Department of Health Sciences, Section of Clinical Pharmacology and Oncology, University of Florence, Viale Pieraccini 6, 50139 Firenze, Italy; mariagrazia.giovannini@unifi.it

The gut, along with its microbiota (MB-gut), is the largest absorption organ and reservoir of bacteria in the human body. The MB-gut is considered a single system whose interactions give rise to responses that affect the functions of the whole body. The central nervous system is in continuous cross-talk with the MB-gut in the so-called MB-gut–brain axis, and many bottom-to-top pathways, activated by MB products, are necessary for the correct development and physiological functions of the brain.

Dysbiosis contributes to many pathological conditions in both the aged and young population. Elucidating how the MB-gut can affect the central nervous system in aging, Alzheimer’s disease, multiple sclerosis and other neurodegenerative pathologies is of the utmost importance. Understanding the interactions between the MB-gut, the enteric system, immune cells, neurons and glia and their implications for host defense, tissue repair and neurodegeneration will be crucial to identifying new actors in the molecular basis of diseases.

In this regard, it is necessary to follow a multidisciplinary approach extended to all the districts and components of the complex MB-gut–brain axis. In particular, analysis of the MB-gut-driven alterations in the neuron–astrocyte–microglia triad will highlight neurodegenerative mechanisms related to differential recruitment/activation of glial cells, improve the knowledge of molecules involved in neuron/glia communication and elucidate MB-gut changes that could prevent and/or delay neurodegeneration.

The present editorial introduces the new Special Issue published by the *International Journal of Molecular Sciences* entitled “The Microbiota–Gut–Brain Axis in Behaviour and Brain Disorders”, which covers this important topic with a collection of six valuable contributions, namely, four original research articles and two reviews.

Liang et al. [1] investigated the anti-constipation effects of *Hemerocallis citrina Baroni* (Daylily, DHC) on gastrointestinal transit, defecation parameters, short-chain organic acids, the gut microbiome, transcriptomes and network pharmacology. The authors demonstrated that the administration of DHC accelerates the defecation frequency of mice and elevates the abundance of some beneficial bacterial taxa while reducing the levels of pathogens in cecal contents. The transcriptomic analysis found more than 700 differentially expressed genes (DEGs) in the colon after DHC intervention, which are mainly involved in the olfactory transduction pathway. The integration of transcriptomics and network pharmacology revealed seven overlapping targets (Alb, Drd2, Igf2, Pon1, Tshr, Mc2r and Nalcn). A qPCR analysis further showed that DHC effectively down-regulates the expression of Alb, Pon1 and Cnr1 in the colon. These results improve the understanding of the anti-constipation effect of DHC, providing a novel integrated perspective of transcriptomes and network pharmacology.

Nuccio et al. [2] examined the impact of social isolation on the intestinal microbiome and metabolome in *Microtus ochrogaster* (prairie vole). Physiological stress causes increased behavioural indicators of anxiety and depression in isolated female prairie voles in comparison with paired prairie voles. Bacterial DNA sequencing at the level of the 16S rRNA gene revealed several differences in gut communities of paired and isolated voles. The authors also found that several taxa associated with host health are prevalent in paired voles, whereas several taxa associated with known pathogens are increased in isolated animals. Similarly, metabolome analyses suggested isolated voles, when compared to paired animals, exhibit differences in metabolites associated with diabetes and colitis. These findings contribute to the understanding of the harmful effects of social isolation, which cause perturbations in the gut microbiome and serum metabolites. 

Sun et al. [3] examined the involvement of gut microbiota in AD by performing behavioural tests, pathological examinations, metagenomics and metabolomics to analyse the gut microbiota and metabolome in APPswe/PS1DE9 (PAP) mice with cognitive decline. They showed that PAP mice, compared to controls, have a different composition of the bacterial communities, showing that the abundances and diversities of the bacterial communities in healthy mice are higher than in PAP mice. The authors demonstrated that PAP mice possess peculiar metabolic phenotypes in their stool, serum and hippocampus, such as alterations in neurotransmitter metabolism, lipid metabolism, aromatic amino acid metabolism, energy metabolism, vitamin digestion and absorption, and bile metabolism. The microbiota–host metabolic correlation analysis suggested that abnormal metabolism may be modulated by some taxa of the gut microbiota. The authors argued that modification of the host metabolism targeting gut microbiota may be a novel and viable strategy for the prevention and treatment of AD.

Chudzik et al. [4] investigated whether treatment with *Lacticaseibacillus rhamnosus JB-1* (JB-1) alters brain metabolite levels and behaviour during continuous exposure to chronic stress in rats. The authors demonstrated that JB-1 dietary supplementation mitigates anxiety; they also showed a significant decrease in glutamine + glutathione in the placebo group compared to the JB-1 bacteria-supplemented group. Furthermore, in placebo rats, they found that the progression of stress caused a decrease in glutamate, glutathione and taurine, while the levels of brain metabolites in the JB-1-supplemented rats were stable throughout the experiment, with only the taurine level decreasing. The authors concluded that the JB-1 bacteria diet may stabilise the levels of stress-related neurometabolites in the rat brain and could prevent the development of anxiety/depressive-like behaviour.

The review by Masanetz et al. [5] proposes the gut–immune–brain axis as the underlying anatomical and functional route leading to depression and other neuropsychiatric symptoms in inflammatory bowel disease (IBD). The authors reviewed the current understanding of IBD pathophysiology and the development of systemic inflammation in IBD. They also shed light on different anatomical routes conveying this inflammation to the CNS and proposed that these routes do not represent passive barriers, but are dynamically regulated and actively contribute to CNS immune activation. The authors then focused on the current knowledge and open questions regarding neuroinflammation in distinct brain regions of preclinical IBD models. In addition, they highlighted the present understanding of neuronal pathology and behavioural impairment in IBD, and how these are linked to CNS immune activation. Finally, they shed light on how the gut microbiota may interfere with different stages of the gut–immune–brain axis to promote neuropsychiatric morbidity in IBD.

The review performed by Lee et al. [6] focuses specifically on diet, physical activity/exercise, substance use and sleep in the context of the emerging adult (EA). Starting from the association between gut microbiota (GM) and mental health, the authors suggest that the period of emerging adulthood may be critical for manipulating and establishing long-term homeostasis of the gut–brain–microbiome (GBM) axis. In addition, they highlight that the extensive changes in the brain during adolescence and early adulthood may function as a nexus in a series of complex, potentially bidirectional causal relationships among genetics, lifestyle factors, mental illnesses and the maturing GM, suggesting that this age group (18–25) should be a separate age category in clinical trials.

These contributions further highlight the importance of the interactions between the MB-gut, the enteric system, immune cells, neurons and glia and their implications for host defense, tissue repair and neurodegeneration. Modification of gut microbiota may be a novel and viable strategy for the prevention and treatment of many neurodegenerative diseases.
